# Flavonoids Inhibit Cancer by Regulating the Competing Endogenous RNA Network

**DOI:** 10.3389/fonc.2022.842790

**Published:** 2022-03-18

**Authors:** Chengshun Li, Xiaolan Li, Ziping Jiang, Dongxu Wang, Liqun Sun, Jiaqi Li, Yang Han

**Affiliations:** ^1^ Laboratory Animal Center, College of Animal Science, Jilin University, Changchun, China; ^2^ State Key Laboratory of Biotherapy, West China Hospital, Sichuan University, Chengdu, China; ^3^ Department of Hand and Foot Surgery, The First Hospital of Jilin University, Changchun, China; ^4^ Department of Pediatrics, First Hospital of Jilin University, Changchun, China

**Keywords:** flavonoids, gastric cancer, miRNA, lncRNA, the competing endogenous RNA network

## Abstract

Flavonoids are present in a wide range of plants. They have been used in the treatment of cancer, but the mechanism underlying this activity is unclear. In recent years, microRNA (miRNA) and long non-coding RNA (lncRNA) levels have been observed to differ between normal tissues and cancer cells, and both types of RNA have been shown to have a role in tumor treatment. In addition, flavonoids have been proven to regulate miRNAs and LncRNAs in the treatment of cancer. The competing endogenous RNA (ceRNA) network is a complex post-transcriptional regulatory mechanism in cells, in which coding and non-coding RNAs competitively bind miRNAs to regulate messenger RNAs (mRNAs). This review focused on the role of the ceRNA network in the treatment of cancer by flavonoids.

## Introduction

Flavonoids are a group of polyphenol compounds, including flavones, flavanols, chalcone and others, with a C6-C3-C6 structure (1). They are widely distributed in nature, mainly in plants and fruit. For instance, chrysin is found in the stems and seeds of plants in the family Bignoniaceae and Pinus bungeana. There is growing evidence that a range of flavonoids have strong pharmacological effects, such as anti-inflammatory, vascular protective, anti-oxidative, and anti-viral properties (2, 3). A study performed that dietary flavonoid intake is associated with a reduced risk of different types of cancer, such as gastric, breast, prostate, and colorectal cancers (4). A few studies have also demonstrated an important role of flavonoids in cancer treatment by mechanisms such as inducing apoptosis and inhibiting proliferation. Flavones, such as vitexin can suppress melanoma cell growth (5), chrysin and luteolin can induced apoptosis in HeLa cells (6, 7). Myricetin, as a member of flavonols, can prevent the incidence of colorectal tumorigenesis and reduce the size of colorectal polyps (8). Flavanonol taxifolin can inhibit the migration and invasion of breast cancer cells (9). These evidences suggest that various flavonoids have positive effects on anti-cancer.

The competing endogenous RNA (ceRNA) hypothesis was initially proposed in 2011 (10). It suggested that various RNAs may regulate each other through microRNAs (miRNAs). Previous studies usually focused only on the unidirectional regulation between miRNA and target genes. It was clear that the mechanism of action of miRNAs involved inhibiting mRNA translation or promoting mRNA degradation (11). The ceRNA hypothesis proposed that RNAs can regulate one another by using the same miRNA response elements (MREs) as a molecular sponge to competitively bind miRNAs (12). This advanced our understanding of the function of non-coding RNAs (ncRNAs). A large part of the human transcriptional genome comprises ncRNAs, and they have a reported role in cancer. Compared with normal tissues, numerous ncRNAs show differential expression in cancer tissues (13). Previous studies have proved that abnormally expressed long ncRNAs (lncRNAs) can promote cancer through the ceRNA network, whereas reversing the expression of LncRNAs can inhibit tumor growth (14–16).

**Figure 1:**
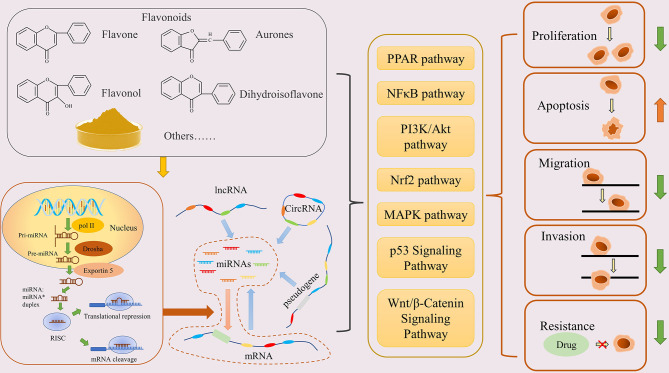
Flavonoids inhibit cancer through the ceRNA network. Flavonoids promote miRNA production, then regulate the ceRNA network, and finally regulate the physiological function of cancer cells *via* various pathways.

In addition, sequencing results have shown that flavonoids, such as galangin, baicalein, chrysin, can change the expression level of mRNAs in tumors, as well as causing the differential expression of miRNAs and LncRNAs (https://www.ncbi.nlm.nih.gov/geo/). These evidences suggest that the anti-cancer mechanism of flavonoids may be related to the regulation of the ceRNA network (**Figure 1**).

## miRNAs and the ceRNA Network

miRNAs are small single-stranded ncRNAs with a length of about 20 nucleotides (nt) (17). After a series of processing steps from the nucleus to the cytoplasm, miRNAs finally mature and assemble with Argonaute (Ago) family proteins to form the RNA-induced silencing complex (RISC) (18, 19). The parts of the target transcript that complement the miRNA sequences are called MREs; they are specifically recognized by miRNAs and guide the RISC to target RNAs, thereby either inhibiting the translation or inducing the degradation of the target mRNA. In humans, the base pairing between miRNAs and their targets is not usually highly specific, which makes the regulatory ability of miRNAs more extensive (20). With the progress of sequencing technology, numerous miRNAs have been characterized and differential changes have been found in various cancers (21). In this context, microRNAs are usually divided into two categories: carcinogenic miRNAs, which are generally highly expressed in tumors, inhibit tumor-suppressor genes, and promote the occurrence and development of tumors, such as miR-675 (22) and miR-501-5p (23); and tumor-suppressor miRNAs, such as miR-199a-3p (24) and miR-101 (25). Notably, the role of miRNAs has tissue specificity. For example, miR-125b can inhibit cell proliferation and induce apoptosis in colon cancer, and participate in tumor drug-resistance in hepatocellular carcinoma (HCC) (26, 27). miRNAs are central to the ceRNA network, through which RNAs can regulate others with similar MRE communication. In addition, a recent study found that the flavonoid scutellarin could alter the miRNA-expression profile of hepatoma cells (28). These findings suggest that regulating miRNA expression is an important mechanism-of-action of flavonoids in cancer treatment.

## LncRNAs as Molecular Sponges in the ceRNA Regulatory Network

LncRNAs are ncRNAs with a length of more than 200 bases, which are transcribed by RNA polymerase II. Although LncRNAs do not encode proteins, they have a variety of biological functions, including gene activation and silencing, alternative splicing, and post-translational modification (29, 30).

On the one hand, LncRNAs can form miRNA precursors through intracellular shear and further progress into mature miRNAs. On the other hand, as an important part of the ceRNA network, LncRNAs are rich in MREs, which allows them to act as a molecular sponge to competitively bind miRNAs and regulate their targets.

A large number of differentially expressed LncRNAs have been found in cancer. Similar to miRNAs, LncRNAs can be divided into cancer-promoting and cancer-inhibiting types. The most common oncogenic LncRNA-*H19* was one of the first to be described (31), and has been proved to be overexpressed in many cancers. *H19* overexpression in bladder cancer cells reduces E-cadherin, thereby promoting cell migration and invasion (32), and promotes the epithelial-to-mesenchymal transition in colorectal cancer by absorbing miR-138 and miR-200a (33). In addition, LncRNAs such as *LncRNA00364*, *TPTEP1*, and *TSLNC8* can inhibit the proliferation and progression of HCC cells (34). Evidence suggests that the abnormal expression of LncRNAs in cancer can be reversed by flavonoids, and has an inhibitory effect on cancer (35). This indicates that flavonoids can regulate the expression of LncRNAs for cancer treatment.

## mRNAs are Both Regulators and Regulated in the ceRNA Network

Because mRNAs are eventually translated into proteins that play biological roles, they often appear to be the main targets of regulation in the ceRNA network; however, they can also participate in the post-transcriptional regulation of other genes, which complicates the crosstalk (36). mRNAs can regulate key genes in carcinogenesis and development in an miRNA-dependent manner. *HMGA2* can increase the expression of *TGFBR3* as a molecular sponge of the let-7a family to promote the occurrence of lung cancer (37). A previous study predicted that *NCALD* may affect drug resistance and prognosis in ovarian cancer by acting as a ceRNA of *CX3CL1* for tumor suppression (38). A large number of reports have shown that flavonoids can regulate mRNAs in tumors to inhibit their progression, and some mRNAs have been proven to be regulated by miRNAs that are affected by flavonoids (39, 40). In conclusion, mRNAs play a prominent role in the anti-cancer effect of flavonoids, and flavonoids can inhibit cancer by regulating mRNAs directly and indirectly.

## Flavonoids Can Inhibit Cancer Through the ceRNAsNetwork

Numerous studies have shown that flavonoids play a positive role in the treatment of cancer (41, 42). There are evidences that flavonoids can inhibit various kinds of cancer in many different ways (43–45). Flavonoids can affect both coding RNAs and ncRNAs in cancer cells, suggesting that the regulation of the ceRNA network is involved in the inhibitory effect of flavonoids on the occurrence and development of cancer. Our previous study suggested that chrysin could promote the apoptosis of gastric cancer cells through the *H19*/miR-Let-7a/*COPB2* axis (16). Furthermore, as a kind of flavonols, quercetin can inhibit proliferation and invasion by up-regulating miR-146a in human breast cancer cells (46), and increase the sensitivity of non-small-cell lung cancer cells to radiotherapy by regulating the miR-16-5p/*WEE1* axis (47). Aside from inhibiting cancer, flavonoids can reverse drug resistance through the ceRNA network.

**Figure 2:**
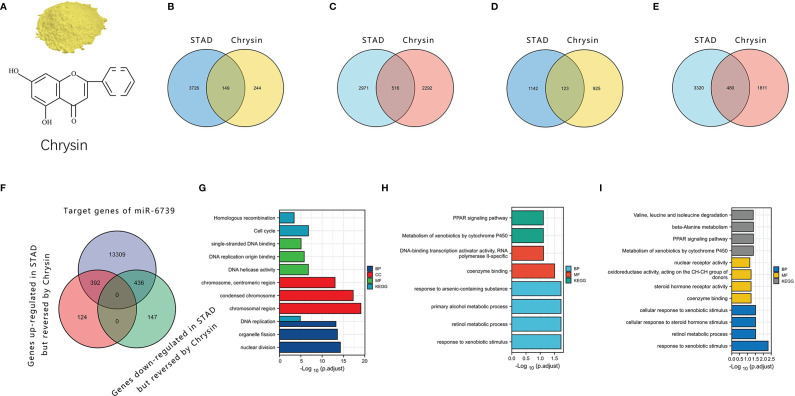
The role of chrysin in gastric cancer through the ceRNA network. **(A)** The structure of chrysin. **(B, C)** LncRNAs and mRNAs that were upregulated in STAD but reversed by chrysin. **(D, E)** LncRNAs and mRNAs that were downregulated in STAD but reversed by chrysin. **(F)** Intersection of the miR6739 target gene and the genes reversed by chrysin in gastric cancer. **(G, H)**. Enrichment for Kyoto Encyclopedia of Genes and Genomes (KEGG) and KEGG Orthology (GO) gene sets of the genes reversed by chrysin. **(I)** Enrichment of KEGG and GO gene sets of the target genes of miR-6739.

We further analyzed the sequencing data of gastric cancer cells treated with chrysin (Gene Expression Omnibus [GEO] accession: GSE181492). The results showed that chrysin could regulate the mRNA expression of nuclear factor kappa B (NFκB), p53, and other signaling pathways, which may be a key link in the complex anticancer effect of chrysin (**Figure 2**). Moreover, we found that some LncRNAs, miRNAs, and mRNAs were expressed abnormally in stomach adenocarcinoma (STAD), but the reverse pattern was seen in gastric cancer cells treated with chrysin. Notably, only miR-6739 was found to have a rescue effect on chrysin in the sequencing results. It was upregulated in gastric cancer and downregulated after chrysin treatment. This indicated that miRNA may play key roles in the chrysin treatment of gastric cancer. According to the prediction of its direct target gene by MREs, chrysin may inhibit gastric cancer *via* miR-6739 through peroxisome proliferator-activated receptor (PPAR) and other pathways.

## Discussion

Existing cancer treatment mainly involves surgical resection supplemented by chemotherapy. Apart from having side-effects for patients, chemotherapy also has an important limitation in terms of drug resistance. However, some of the members of flavonoids can reverse drug resistance of cancers by various ways. For example, baicalein can increase cisplatin sensitivity of A549 lung adenocarcinoma cells (48). Luteolin can enhance chemosensitivity of breast cancer through the Nrf2-Mediated pathway (49). Quercetin can increase the chemosensitivity of breast cancer to doxorubicin *Via* PTEN/Akt pathway (50). Therefore, flavonoids might can be used to reduce the side-effects of chemotherapy drugs and increase the sensitivity of tumors. Studies have shown that abnormal miRNA and LncRNA expression can cause drug resistance in cancer (51, 52). The flavonoids mentioned above may reverse drug resistance with the help of the ceRNA network; flavonoids may therefore be used to reduce the side-effects of chemotherapy drugs and increase the sensitivity of tumors, in order to treat cancer more safely and effectively. In addition, there is evidence that quercetin, chrysin, luteolin and other flavonoids contribute to wound healing (53), which may aid patients’ postoperative recovery. These make flavonoid drugs have a better prospect in the combination of other drugs in the treatment of cancer.

In conclusion, the ceRNA network plays an important role in cancer treatment with flavonoids. Flavonoids can regulate cancer-related genes *via* the ceRNA network. Moreover, the ceRNA network can have additional effects that may help patients with treatment and recovery. These findings show that the ceRNA network offers new prospects for the use of flavonoids in anti-cancer treatment.

## Author Contributions

CL and DW wrote the manuscript. CL, XL, ZJ, LS, YH, and JL collected the references and prepared figures. All authors reviewed the manuscript. All authors contributed to the article and approved the submitted version.

## Funding

This work was supported by Natural science Foundation of the Jilin province (20210101310JC) and Chinese Postdoctoral International Exchange Program.

## Conflict of Interest

The authors declare that the research was conducted in the absence of any commercial or financial relationships that could be construed as a potential conflict of interest.

## Publisher’s Note

All claims expressed in this article are solely those of the authors and do not necessarily represent those of their affiliated organizations, or those of the publisher, the editors and the reviewers. Any product that may be evaluated in this article, or claim that may be made by its manufacturer, is not guaranteed or endorsed by the publisher.
